# A bioinformatic search for correspondence between differentially expressed genes of domestic versus wild animals and orthologous human genes altering reproductive potential

**DOI:** 10.18699/VJGB-22-13

**Published:** 2022-02

**Authors:** M.P. Ponomarenko, I.V. Chadaeva, P.M. Ponomarenko, A.G. Bogomolov, D.Yu. Oshchepkov, E.B. Sharypova, V.V. Suslov, A.V. Osadchuk, L.V. Osadchuk, Yu.G. Matushkin

**Affiliations:** Institute of Cytology and Genetics of the Siberian Branch of the Russian Academy of Sciences, Novosibirsk, Russia; Institute of Cytology and Genetics of the Siberian Branch of the Russian Academy of Sciences, Novosibirsk, Russia; Institute of Cytology and Genetics of the Siberian Branch of the Russian Academy of Sciences, Novosibirsk, Russia; Institute of Cytology and Genetics of the Siberian Branch of the Russian Academy of Sciences, Novosibirsk, Russia; Institute of Cytology and Genetics of the Siberian Branch of the Russian Academy of Sciences, Novosibirsk, Russia; Institute of Cytology and Genetics of the Siberian Branch of the Russian Academy of Sciences, Novosibirsk, Russia; Institute of Cytology and Genetics of the Siberian Branch of the Russian Academy of Sciences, Novosibirsk, Russia; Institute of Cytology and Genetics of the Siberian Branch of the Russian Academy of Sciences, Novosibirsk, Russia; Institute of Cytology and Genetics of the Siberian Branch of the Russian Academy of Sciences, Novosibirsk, Russia; Institute of Cytology and Genetics of the Siberian Branch of the Russian Academy of Sciences, Novosibirsk, Russia

**Keywords:** human, reproductive potential, animal model of human disease, domestication, RNA-Seq, most recent common ancestor, человек, репродуктивный потенциал, модель болезни человека с использованием животных, доместикация, RNA-Seq, ближайший общий предок

## Abstract

One of the greatest achievements of genetics in the 20th century is D.K. Belyaev’s discovery of destabilizing selection during the domestication of animals and that this selection affects only gene expression regulation (not gene structure) and inf luences systems of neuroendocrine control of ontogenesis in a stressful environment. Among the experimental data generalized by Belyaev’s discovery, there are also f indings about accelerated extinc tion of testes’ hormonal function and disrupted seasonality of reproduction of domesticated foxes in comparison with their wild congeners. To date, Belyaev’s discovery has already been repeatedly conf irmed, for example, by independent observations during deer domestication, during the use of rats as laboratory animals, after the reintroduction of endangered species such as Przewalski’s horse, and during the creation of a Siberian reserve population of the Siberian grouse when it had reached an endangered status in natural habitats. A genome-wide comparison among humans, several domestic animals, and some of their wild congeners has given rise to the concept of self-domestication syndrome, which includes autism spectrum disorders. In our previous study, we created a bioinformatic model of human self-domestication syndrome using differentially expressed genes (DEGs; of domestic animals versus their wild congeners) orthologous to the human genes (mainly, nervous-system genes) whose changes in expression affect reproductive potential, i. e., growth of the number of humans in the absence of restrictions caused by limiting factors. Here, we applied this model to 68 human genes whose changes in expression alter the reproductive health of women and men and to 3080 DEGs of domestic versus wild animals. As a result, in domestic animals, we identif ied 16 and 4 DEGs, the expression changes of which are codirected with changes in the expression of the human orthologous genes decreasing and increasing human reproductive potential, respectively. The wild animals had 9 and 11 such DEGs, respectively. This difference between domestic and wild animals was signif icant according to Pearson’s χ2 test (p < 0.05) and Fisher’s exact test (p < 0.05). We discuss the results from the standpoint of restoration of endangered animal species whose natural habitats are subject to an anthropogenic impact.

## Abstract (ru)

Одним из крупнейших достижений генетики ХХ в. стало открытие Д.К. Беляевым дестабилизирующего отбора при одомашнивании животных, который затрагивает регуляцию экспрессии генов, но не их структуру, и влияет на системы нейроэндокринного контроля онтогенеза при стрессовом воздействии окружающей среды. Среди экспериментов, результаты которых были обобщены этим открытием, были также наблюдения ускоренного угасания гормональной функции семенников и нарушения сезонности размножения одомашненных лисиц по сравнению с дикими сородичами. На сегодняшний день открытие Д.К. Беляева уже многократно подтверждено независимыми наблюдениями при одомашнивании, например, оленя, использовании крыс как лабораторных животных, восстановлении таких исчезающих видов, как лошадь Пржевальского, а также при создании резервной популяции дикуши, оказавшейся на грани исчезновения в естественных местах обитания. В результате сравнения геномов человека, ряда домашних животных и некоторых их диких сородичей был введен термин «синдром самодоместикации», в симптомы которого включены расстройства аутистического спектра. Ранее мы создали биоинформатическую модель синдрома самодоместикации человека с использованием дифференциально экспрессируемых генов (ДЭГ) домашних животных по сравнению с дикими сородичами, ортологичных генам преимущественно нервной системы человека, чьи изменения экспрессии могут влиять на репродуктивный потенциал, т. е. рост численности людей при отсутствии ограничений со стороны лимитирующих факторов. В настоящей работе мы применили эту модель к 68 генам человека, изменения экспрессии которых влияют на репродуктивное здоровье женщин и мужчин, и к 3080 ДЭГ животных. Обнаружено 16 и 4 ДЭГ домашних животных, изменения экспрессии которых являются сонаправленными изменениям экспрессии генов-ортологов человека, соответственно понижающим и повышающим его репродуктивный потенциал, тогда как у диких животных было 9 и 11 таких ДЭГ. Это различие было достоверно по критерию χ2 Пирсона ( p < 0.05) и точному критерию Фишера ( p <0.05). Полученный результат обсуждается в связи с восстановлением видов животных, исчезающих под антропогенной нагрузкой.

## Introduction

One of the greatest achievements of genetics in the 20th century was D.K. Belyaev’s discovery of destabilizing selection during the domestication of animals and his finding that this selection affects the regulation of gene expression (i. e., specificity and level of expression) but not gene structure. In this context, destabilizing selection directly or indirectly affects systems of neuroendocrine control of ontogenesis when preexisting stress factors strengthen or new ones emerge in the environment: “In a genetic and biochemical sense, what may be selected for are changes in the regulation of genes – that is, in the timing and the amount of gene expression rather than changes in individual structural genes. Selection having such an effect is called by me destabilizing selection. The selection becomes destabilizing when it affects, directly or indirectly, the systems of neuroendocrine control of ontogenesis. This seems always to be the case when some new stressful factors appear in the environment, or when stresses usual for the species increase in strength.” (Belyaev, 1979, p. 307).

This discovery is the result of many years of unique experiments on the domestication of the mink (Belyaev et al., 1972) and fox (Belyaev et al., 1975) as well as the mouse as a laboratory model of human cancer (Belyaev, Gruntenko, 1972). In these experiments, there were findings about accelerated extinction of testes’ hormonal function (Osadchuk et al., 1978a) and disturbances in reproduction seasonality (Osadchuk et al., 1978b) of domesticated foxes versus wild foxes; these experiments were conducted with the participation of a coauthor of the present study.

Subsequent comparative analysis of reproductive indices of domesticated foxes versus wild ones (taken as the norm) revealed decreases in the activity indicators of the female endocrine system (Osadchuk, 1992a), in sexual activity of first-year males (Osadchuk, 1992b, 2006), in embryonic gonad mass, and in developmental heterochrony of their pituitarytesticular axis (Osadchuk, 1998) as evidence of destabilizing selection during the domestication of animals (Belyaev, 1979). Additionally, in a laboratory model of animal domestication involving outbred rat strains, a delay in puberty was independently documented in males of a tame strain compared to an aggressive strain (Prasolova et al., 2014). The results of a comparison among the genomes of humans, numerous domestic animals, and some of their wild congeners have been generalized by the term “self-domestication syndrome”, the symptoms of which include autism spectrum disorders (Theofanopoulou et al., 2017), although the idea of human self-domestication is still subject to debate (Del Savio, Mameli, 2020) to this day.

Following a trend in the postgenomic era of life sciences (Qian et al., 2021), we have created a bioinformatic model of self-domestication syndrome using differentially expressed genes (DEGs) – of domestic animals versus their wild congeners – that are orthologous to human genes associated with rheumatoid arthritis (Klimova et al., 2021) and with reproductive potential (Vasiliev et al., 2021), i. e., with an increase in the number of humans when there are no restrictions caused by limiting factors (Chapman, 1931; Pianka, 1976).

In the present work, we analyzed 68 human genes whose expression changes affect the reproductive health of women (Chadaeva et al., 2018) and men (Ponomarenko et al., 2020). The results are discussed in terms of restoration of animal species that are disappearing under anthropogenic pressure (Esmaeili et al., 2019).

## Materials and methods

The analyzed human genes. We analyzed 68 human genes in the promoters of which we have previously evaluated candidate SNP markers of changes in the reproductive health of women (Chadaeva et al., 2018) and men (Ponomarenko et al., 2020); the examples are presented in Table 1, and complete descriptions – in Supplementary Material


Supplementary Material is available in the online version of the paper: http://vavilov.elpub.ru/jour/manager/files/Suppl_Ponomarenko_Engl.pdf 


**Table 1.**
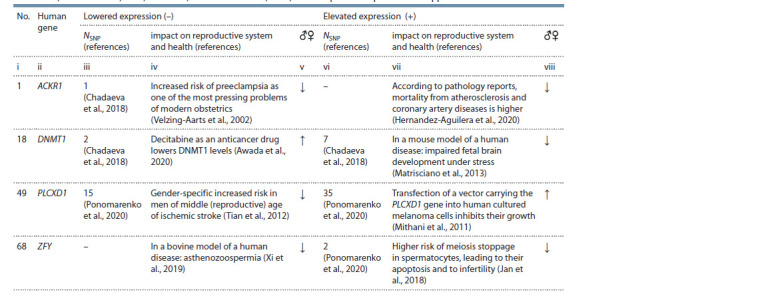
Examples of the 68 studied human genes for which a signif icant effect of an SNP(s) in the binding site for TATA-binding protein (TBP) on the aff inity of TBP for the promoter of these genes has been previously documented, as have the effects on the levels of their expression and corresponding changes in the reproductive system of women (Chadaeva et al., 2018) and men (Ponomarenko et al., 2020). The complete list is provided in Supplemental Material Notе. No. is the ID number of a gene in the full list, sorted alphabetically in Supplementary Material. NSNP is the number of candidate SNP markers that signif icantly reduce or increase the aff inity of TBP for a promoter of a gene (Chadaeva et al., 2018; Ponomarenko et al., 2020), thus decreasing (–) or increasing (+) its expression (Mogno et al., 2010; Ponomarenko et al., 2010); impact on reproductive system and health: deterioration (↓) or improvement (↑). Genes: ACKR1 – atypical chemokine receptor 1; DNMT1 – DNA methyltransferase 1; PLCXD1 – phosphatidylinositol-specif ic phospholipase CX domain – containing 1; ZFY – Y- linked zinc f inger protein.

For instance, in the promoter of the human ACKR1 gene (atypical chemokine receptor 1), we previously found SNP rs2814778, which lowers the affinity of TATA-binding protein (TBP) for this promoter (Chadaeva et al., 2018), thereby (Mogno et al., 2010) lowering the expression of this gene (see Table 1, column iii, NSNP = 1). This finding is consistent with independent clinical data on patients carrying rs2814778 (Michon et al., 2001; Nalls et al., 2008), and therefore we proposed rs2814778 as a candidate SNP marker of preeclampsia as one of the most pressing problems of modern obstetrics (Velzing-Aarts et al., 2002), which worsens the reproductive health of women (Chadaeva et al., 2018), as indicated by the down arrow (↓) in column v of Table 1. On the other hand, according to pathology reports (Hernandez-Aguilera et al., 2020), an excess of the ACKR1 protein contributes to increased human mortality from atherosclerosis and other coronary artery diseases (see Table 1, column vii), thus reducing human reproductive potential (see Table 1, column viii).

Another example of a gene studied by us earlier (Ponomarenko et al., 2020), the decrease and increase in expression of which impair the reproductive system of humans, is ZFY (located on the Y chromosome) encoding a protein with a zinc finger (see Table 1).

In addition, we previously found two candidate SNP markers, rs758026532 and rs772821225, in the promoter of DNMT1 encoding human DNA methyltransferase 1 – that reduce DNMT1 expression (Chadaeva et al., 2018), as does anticancer drug decitabine (Awada et al., 2020), thereby increasing the reproductive potential of people (see Table 1, column v, symbol “↑”). Besides, in the promoter of this gene, we previously found seven candidate SNP markers of DNMT1 overexpression (Chadaeva et al., 2018), which, according to a mouse model of a human disease (Matrisciano et al., 2013), can cause epigenetic aberrations of fetal brain development under the influence of stressors, thus impairing the human reproductive system (see Table 1, column viii, symbol “↓”).

Finally, in Table 1, readers can see that the previously studied (Ponomarenko et al., 2020) PLCXD1 gene (phosphatidylinositol- specific phospholipase CX domain-containing 1) represents a diametrically opposite situation (see Table 1, symbols “↓” and “↑” in columns v and viii, respectively). Indeed, underexpression of this gene is a risk factor for stroke in men of reproductive age (Tian et al., 2012), whereas its overexpression improves human reproductive potential by suppressing the progression of melanomas: some of the deadliest human malignant tumors (Mithani et al., 2011).

As done for the genes ACKR1, DNMT1, PLCXD1, and ZFY above, Supplementary Material describes all 68 human genes analyzed in the present study.

The studied DEGs of domestic versus wild animals. A total of 3080 DEGs of domestic versus wild animals were analyzed here, which are freely available in the PubMed database (Lu, 2011), as described in Table 2 and characterized by examples in Table 3. At the same time, according to (Klimova et al., 2021; Vasiliev et al., 2021), here, RNA-Seq data were examined in accordance with one of the oldest (Samet, 1985), widely used (Sun et al., 2008; Morozova et al., 2020; Hakizimana et al., 2021), and fundamental (Zhang et al., 2021) concepts of phylogenetic analysis – “most recent common ancestor” (Samet, 1985). In this regard, domestic animals and their wild relatives were studied by means of oppositely directed equivalent changes in gene expression in comparison with their unknown most recent common ancestor.

**Table 2.**
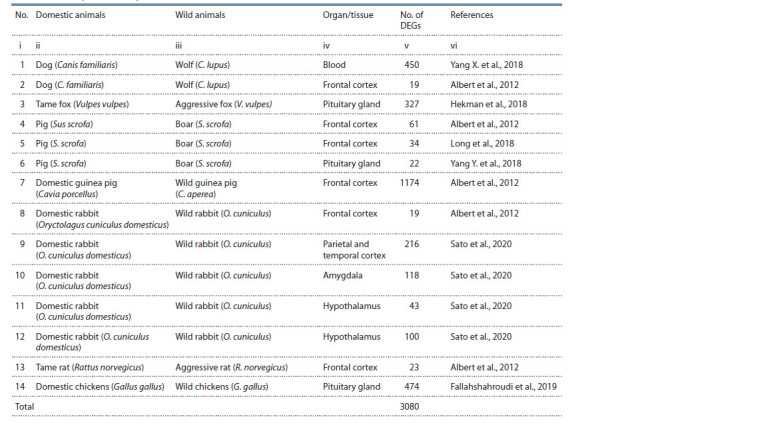
The analyzed RNA-Seq data on DEGs of domestic vs wild animals available in the PubMed database (Lu, 2011)

For example, the Ckbl gene (creatine kinase B-like protein) was characterized in column v of Table 1 by a positive score of 4.33 log2 units of relative expression in the blood of dogs (Canis familiaris) versus wolves (C. lupus), as reported by (Yang X. et al., 2018). Therefore, dogs and wolves respectively show increased and decreased expression of this gene as compared to their most recent common ancestor (see Table 3, columns vii and viii). Likewise, a negative score of (–1.55) on the relative expression of Adm (adrenomedullin) in the dog’s frontal cortex as compared to the wolf (see Table 3, column v) corresponds to decreased and increased expression of this gene in this part of the brain during divergence from their most recent common ancestor (see Table 3, columns vii and viii). A total of 450 DEGs in the blood (Yang X. et al., 2018) and 19 DEGs in the frontal cortex (Albert et al., 2012) of dogs and wolves (see Table 2, column v) were characterized in this way.

**Table 3.**
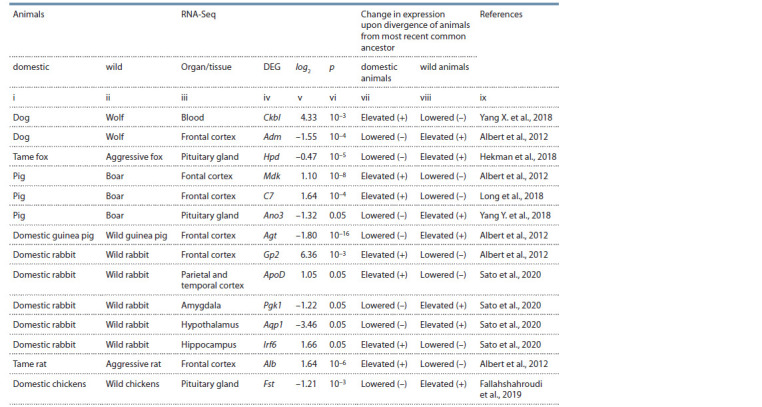
Examples of the studied DEGs of domestic vs wild animals. These DEGs are collectively characterized in Table 2 Notе. log2 – expression in domesticated relative to wild animals (in log2 units); p – statistical signif icance as determined by the authors cited in column ix. Genes: Ckbl – creatine kinase B-like protein; Adm – adrenomedullin; Hpd – 4-hydroxyphenylpyruvate dioxygenase; Mdk – midkine; C7 – component 7 of the complement system of innate immunity; Ano3 – anoctamin 3; Agt – angiotensinogen; Gp2 – glycoprotein 2; ApoD – apolipoprotein D; Pgk1 – phosphoglycerate kinase 1; Aqp1 – aquaporin 1; Irf6 – interferon regulatory factor 6; Alb – albumin; Fst – follistatin.

The score of (–0.47) on the differential expression of the Hpd gene, which encodes 4-hydroxyphenylpyruvate dioxygenase, in the pituitary gland of tame versus aggressive foxes Vulpes vulpes (Hekman et al., 2018) denotes respectively decreased and increased expression of this gene during divergence from their most recent ancestor (see Table 3).

In addition, positive scores on relative expression of genes Mdk (Albert et al., 2012) and C7 (Long et al., 2018) (respectively encoding midkine and component 7 of the complement system of innate immunity) in the frontal cortex of the pig (Sus scrofa) as compared to the boar (S. scrofa) indicates their higher expression in the pig than in the boar when these species diverged from their most recent common ancestor (see Table 3). On the contrary, the negative score of (–1.32) for the Ano3 gene in the pituitary gland of the pig compared to the boar (Yang Y. et al., 2018) denotes respectively a deficiency and an excess of anoctamin 3 (encoded by this gene) in this part of the brain when these species diverged from their most recent common ancestor (see Table 3).

Accordingly, a negative score on the differential expression of the Agt gene (angiotensinogen) in the frontal cortex of domestic guinea pigs Cavia porcellus relative to wild guinea pigs C. aperea (Albert et al., 2012) corresponds to decreased and increased expression of this gene as these animals diverged from their most recent common ancestor (see Table 3, columns v, vii, and viii). Table 3 provides similar examples of description for some of the 3080 DEGs of domestic animals versus their wild congeners, as investigated in this work (groups of all genes are described in Table 2).

A search for orthologous genes of humans and animals. For each analyzed DEG of domestic animals versus their wild congeners (see Tables 2 and 3), an orthologous gene was sought among all the 68 studied human genes (see Table 1 and Supplemental Material). If no such orthologous human gene was found, then the animal DEG in question was excluded from further analysis. Otherwise, we collated the effects of codirected changes in the expression of the found orthologous genes on the reproductive potential of humans (see Table 1 and Supplemental Material, columns v and viii) with expression changes during the emergence of a domesticated species or during preservation of the wild species of the respective animal in the microevolution of their most recent common ancestor (see Table 3, columns vii and viii). For example, the Apoa1 gene (apolipoprotein A1) is characterized by a negative score of (–3.2) on differential expression in domestic versus wild guinea pigs (Albert et al., 2012), indicating decreased and increased expression of this gene, respectively, in the process of their divergence from their most recent common ancestor (Table 4, columns ii, iv, and vi). Accordingly, underexpression of a human orthologous gene, APOA1, was clinically associated with a predisposition to cognitive disorders (Peng et al., 2017), whereas its overexpression correlates with infertility in women (Manohar et al., 2014), as illustrated in columns vii and ix of Table 4. Thus, a deficiency and excess of APOA1 in humans impair the reproductive system of humans (see Table 4, columns viii and x).

**Table 4.**
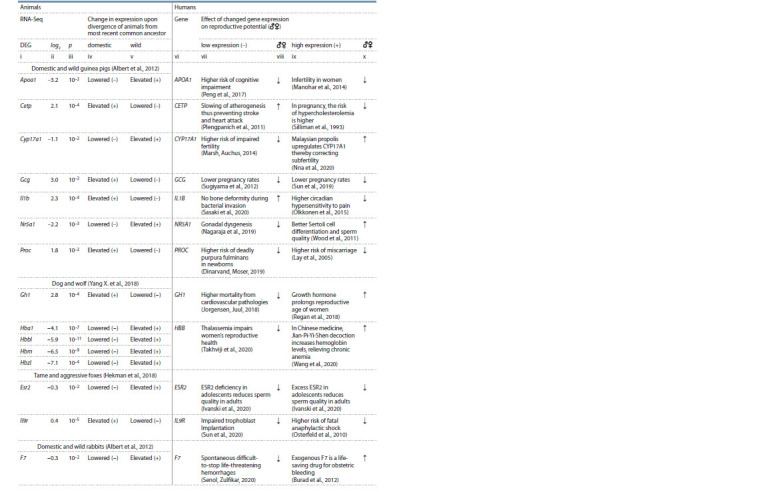
A comparison between the effects of expression changes of human orthologous genes on reproductive potential and expression changes during the divergence of domestic and wild animals from their most recent common ancestor

**Table 4end.**
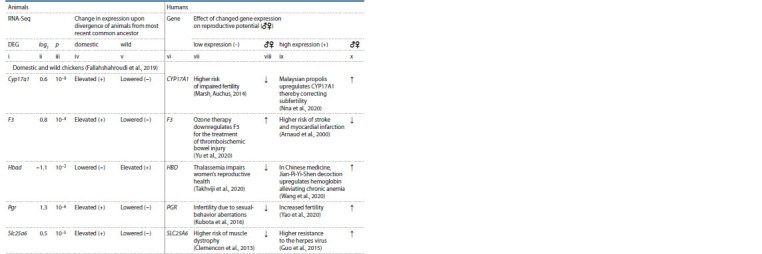
Table 4 (end) Notе. See the footnote of Table 3. Genes: Apoa1 – apolipoprotein A1; Cetp – cholesteryl ester transfer protein; Cyp17a1 – steroid 17α-monooxygenase; Gcg – glucagon; Il1b – interleukin 1β; Nr5a1 – steroidogenic factor 1; F3, F7, and Proc – blood coagulation factors III, VII, and XIV, respectively; Gh1 – growth hormone; HBD, Hba1, Hbad, Hbbl, Hbm, and Hbz1 are hemoglobin subunits δ, α1, αD, β-like, μ, and ζ1, respectively; Esr2 – estrogen receptor 2; Il9r – interleukin 9 receptor; Pgr – progesterone receptor; Slc25a6 – mitochondrial solute transporter.

In the present study, within the framework of the previously proposed bioinformatic model of human diseases involving DEGs of domestic versus wild animals (Klimova et al., 2021; Vasiliev et al., 2021), all of the above means that the expression changes of Apoa1 during the divergence of domestic and wild guinea pigs from their most recent common ancestor correspond to a negative impact of expression changes of the human orthologous gene APOA1 on human reproductive potential.

Similarly, the CETP gene encoding cholesteryl ester transfer protein is overexpressed in hypercholesterolemia of pregnancy (Silliman et al., 1993), thereby impairing the reproductive health of women (see Table 4, columns ix and x). The excess of CETP in humans is consistent with an excess of Cetp in the domestic guinea pig during its divergence from the most recent common ancestor with the wild guinea pig (Albert et al., 2012), as shown in Table 4 (columns ii and iv). By contrast, a CETP deficiency in humans is a clinically proven marker of slowing atherogenesis as well as lower risks of stroke and myocardial infarction (Plengpanich et al., 2011); these correlations can be regarded as factors increasing human reproductive potential (see Table 4, columns vii and viii). CETP downregulation in humans is consistent with Cetp downregulation in the wild guinea pig when it diverged with the domestic guinea pig from the most recent common ancestor (Albert et al., 2012) (see Table 4, column v).

Finally, the human CYP17A1 gene produces steroid 17α-monooxygenase, underexpression of which impairs fertility in humans (Marsh, Auchus, 2014), thereby reducing their reproductive potential, as displayed in Table 4. The deficiency of CYP17A1 in humans is consistent with the deficiency of Cyp17a1 in the domestic guinea pig (Albert et al., 2012) and in wild chickens Gallus gallus (Fallahshahroudi et al., 2019) when domestic and wild forms of these animals diverged from their respective most recent common ancestors (see Table 4, columns ii and iv). On the contrary, a CYP17A1 excess in humans overcomes subfertility (Nna et al., 2020), thus increasing human reproductive potential (see Table 4, columns ix and x). This influence is consistent with higher expression of the orthologous Cyp17a1 gene in the wild guinea pig and domestic chicken as compared with this gene’s expression during their microevolution from the corresponding most recent common (see Table 4).

In Table 4, the reader can find similar descriptions for all the human and animal orthologous genes that we identified among the 68 human genes under study (see Table 1 and Supplemental Material) and among the 3080 DEGs of domestic animals versus their wild congeners (see Tables 2 and 3). In this context, it is noteworthy that because of the concept of “divergence from the most recent common ancestor,” it was possible to compare phenotypic manifestations of increased and decreased expression of human genes (see Table 1, columns v and viii; Table 4, columns viii and x) with changes in the expression of respective orthologous genes in domestic and wild animals as they diverged from their most recent common ancestor (see Table 3, columns v and vi; Table 4, columns iv and v).

Knowledge base PetDEGsDB on human diseases as candidate symptoms of self-domestication syndrome. Identified here as the main finding, the matches – between the effects of changed expression of human genes on human reproductive potential and expression changes of orthologous animal genes during the divergence of domestic and wild animals from their most recent common ancestors – were compiled into a flat text Excel-compatible file and were finally transformed in the MariaDB 10.2.12 Web environment (MariaDB Corp AB, Espoo, Finland) into a knowledge base, named PetDEGsDB, on human diseases that are candidates for self-domestication syndrome (Vasiliev et al., 2021). This knowledge base is freely available at https://www.sysbio.ru/domestic-wild.

Statistical analysis. The correspondences (see Table 4) between the phenotypic manifestations of codirected changes in the expression of orthologous genes of humans and animals were summarized in a standard Fisher 2×2 table represented by intersections of the rows “domestic animals” and “wild animals” (Table 5, columns iii and iv). This Fisher 2×2 table was analyzed using the Statistica package (Statsoft™, Tulsa, USA); its operating mode was chosen via the sequence of commands Statistics → Nonparametrics → 2×2 Table”, which enabled us to perform a binomial distribution analysis, Fisher’s exact test, and Pearson’s χ2 test (see Table 5, columns v, vi, vii, and viii).

## Results and discussion

In this work, we examined 68 human genes (see Table 1 and Supplemental Material) and 3080 DEGs of domestic animals versus wild congeners (see Tables 2 and 3), which are described in the “Materials and methods” section. As a result of the technique described in the subsection “A search for orthologous genes of humans and animals” (Materials and methods), 20 animal DEGs were found that turned out to be orthologous to the studied human genes, as presented in Table 4 and described in the “Materials and methods,” with human genes APOA1, CETP, and CYP17A1 as examples. Let us review the identified orthologous genes of humans and animals.

The human CGC gene codes for glucagon; both a deficiency (Sugiyama et al., 2012) and an excess (Sun et al., 2019) of this protein are clinically proven markers of a reduced pregnancy rate and hence impairment of the reproductive system in humans (see Table 4). Upregulation and downregulation of glucagon in humans are consistent with increased and decreased expression of Gcg in domestic and wild guinea pigs (Albert et al., 2012) during their divergence from their most recent common ancestor.

The IL1B gene codes for interleukin 1β. An excess of this interleukin increases circadian sensitivity to pain (Olkkonen et al., 2015), thereby reducing human reproductive potential (see Table 4). By contrast, IL1B deficiency prevents bone deformation during bacterial invasion (Sasaki et al., 2020), thus expectedly increasing human reproductive potential (see Table 4). The excess and deficiency of IL1B in humans are the expression changes codirected with the upregulation and downregulation of Il1b in the wild guinea pig during its divergence with the domestic guinea pig from a common ancestor (Albert et al., 2012).

The NR5A1 gene encoding human steroidogenic factor 1 is characterized by underexpression in gonadal dysgenesis (Nagaraja et al., 2019), which reduces human reproductive potential (see Table 4), whereas overexpression of the NR5A1 protein improves sperm quality (Wood et al., 2011). Both the NR5A1 deficiency and excess in humans are consistent with the decreased and increased expression of Nr5a1 in the domestic guinea pig in the process of divergence with the wild guinea pig from a common ancestor (Albert et al., 2012).

The PROC gene represents human coagulation factor XIV, a deficiency of which in neonates can cause deadly purpura fulminans (Dinarvand, Moser, 2019), whereas its overexpression increases miscarriage risk (Lay et al., 2005). These alterations of PROC expression are in agreement with the decreased and increased expression of Proc in wild and domestic guinea pigs (Albert et al., 2012) during their microevolution (see Table 4).

The GH1 gene codes for growth hormone, which increases the reproductive potential of women (Regan et al., 2018). The excess of GH1 in humans is similar to the excess of Gh1 in dogs (C. familiaris) when compared to the most recent common ancestor of dogs and wolves (C. lupus) (Yang X. et al., 2018). GH1 deficiency increases human mortality from cardiovascular disease (Jorgensen, Juul, 2018) in line with Gh1 deficiency in wolves during their microevolution.

Genes HBB and HBD encode hemoglobin subunits β and δ. Their deficiency is associated with thalassemia, a contributing factor of poor reproductive potential in women (Takhviji et al., 2020). Human hemoglobin deficiency is consistent with hemoglobin underexpression in dogs (Yang X. et al., 2018) and domestic chickens (Fallahshahroudi et al., 2019) when compared with the most recent common ancestors for their wild counterparts (see Table 4). Conversely, an excess of hemoglobin in humans is in agreement with overexpression of hemoglobin in wolves and wild chickens (see Table 4).

The human ESR2 gene (estrogen receptor 2) – both in the case of underexpression in adolescents and in the case of its overexpression in this segment of the population – was associated with decreased sperm quality in adults (Ivanski et al., 2020). These alterations of its expression in humans are consistent with those of an orthologous gene, Esr2, in tame and aggressive foxes (Hekman et al., 2018) during their microevolution (see Table 4).

The IL9R gene encodes human interleukin 9 receptor, the deficiency of which disrupts trophoblast implantation (Sun et al., 2020), whereas its excess contributes to deadly anaphylactic shock (Osterfeld et al., 2010). The upregulation and downregulation of this receptor in humans are consistent with increased and decreased expression of the Il9r gene in tame and aggressive foxes (Hekman et al., 2018) as they diverged from their most recent common ancestor (see Table 4).

The F7 gene encodes proconvertin. Its recombinant activated form is used as an emergency life-saving modality against obstetric bleeding (Burad et al., 2012). Upregulation of F7 in humans is consistent with that of its ortholog in wild rabbits in the process of divergence with domestic rabbits from a common ancestor (Albert et al., 2012). A proconvertin deficiency accompanies spontaneous life-threatening bleeding (Senol, Zulfikar, 2020) and is consistent with F7 deficiency in domestic rabbits (see Table 2).

The F3 gene (thromboplastin) is overexpressed in stroke and myocardial infarction (Arnaud et al., 2000) and thus may reduce human reproductive potential (see Table 4). An excess of F3 in humans is consistent with an excess of F3 in domestic chickens (Fallahshahroudi et al., 2019). On the other hand, thromboplastin deficiency contributes to an increase in human reproductive potential (Yu et al., 2020), in agreement with F3 deficiency in wild chickens during their divergence with domestic chickens from the most recent common ancestor.

The PGR gene codes for progesterone receptor. A human disease model based on Pgr knockout rats features infertility due to impaired sexual behavior (Kubota et al., 2016). PGR deficiency in humans is codirected with Pgr deficiency in wild chickens during their divergence from a common ancestor with domestic chickens (Fallahshahroudi et al., 2019). A human fertility model based on ewes revealed a positive correlation between Pgr and fertility (Yao et al., 2020). Upregulation of PGR in humans is consistent with Pgr overexpression in domestic chickens as a consequence of their selection by humans for egg production (see Table 4).

The SLC25A6 gene encodes human steroidogenic factor 1. Its overexpression correlates with resistance to the herpes virus (Guo et al., 2015), in line with Slc25a6 overexpression in domestic chickens compared to their most recent common ancestor with wild chickens (Fallahshahroudi et al., 2019). An SLC25A6 deficiency is accompanied by an increased risk of muscle dystrophy (Clemencon et al., 2013) in agreement with the Slc25a6 underexpression in wild chickens as compared to their most recent common ancestor with domestic chickens selected for muscle growth by humans.

All the results of this study are summarized in Table 5, where we present domestic animals’ 16 and 4 DEGs the changes in expression of which are consistently codirected with changes in the expression of the orthologous genes in humans that respectively decrease and increase human reproductive potential. By contrast, in the wild animals, there were 9 and 11 such DEGs, respectively (almost equal numbers of oppositely acting DEGs). This difference between wild and domestic animals is statistically significant according to Pearson’s χ2 test (p < 0.05) and Fisher’s exact test (p < 0.05). Finally, the binomial distribution analysis ( p < 0.01) indicates that the anthropogenic living conditions of animals during their domestication usually alter gene expression in a direction corresponding to the expression changes of human orthologous genes that decrease reproductive potential.

On the contrary, microevolution of wild animals in a natural habitat has changed the expression of genes equally often in the directions that either decrease or increase reproductive potential, judging from expression changes of respective human orthologous genes (binomial distribution: p > 0.4). This finding is in agreement with the generally accepted choice of the wild type as the norm.

While discussing this result, we should note, first of all, that in laboratory animal models of human diseases, DEGs are usually detected in inbred strains having symptoms of a disease in comparison with outbred strains as the norm (Fedoseeva et al., 2019).

Nevertheless, in the literature, we were unable to find unequivocal evidence that codirected changes in the expression of orthologous genes cause similar pathologies in humans and animals, probably owing to different genetic contexts of these changes in different species.

Among parameters of the harmful anthropogenic impact on animal populations, a decrease in their effective size is often mentioned, which promotes their inbreeding, which in turn negatively correlates with sperm quality, for example, in the domestic cat Felis catus (Pukazhenthi et al., 2006), deer Cervus elaphus (Gomendio et al., 2007), and finch Taeniopygia guttata (Forstmeier et al., 2017) as well as in Mexican wolves (Canis lupus baileyi), which disappeared from the wild in the 20th century and exist only as part of a program for their restoration and reintroduction into their former habitats (Asa et al., 2007).

When endangered cranes Grus americana are reintroduced, a high degree of inbreeding of their ex situ population (~400 individuals) delays the onset of reproduction, and as a consequence, decreases egg production; this problem is expected to be overcome by sperm cryopreservation and artificial insemination (Songsasen et al., 2019).

For the feline family Felidae, sperm cryopreservation and artificial insemination have already been successfully implemented for the reintroduction of the endangered wild cat Prionailurus bengalensis euptilurus (Amstislavsky et al., 2018). The creation of protected areas for natural habitats of the Amur tiger Panthera tigris altaica has contributed to the restoration of its population (Xiao et al., 2016). Due to an anthropogenic reduction in the geographic range of the Florida cougar Puma concolor coryi, only ~20 individuals are left. On the basis of theoretical populational calculations (Hedrick, 1995), individuals of the closely related Texas cougar P. concolor couguar were transported to restore this species, thereby ensuring the success of the reintroduction (Hedrick, 2010).

Crossing of subspecies has facilitated the reintroduction of Przewalski’s horses Equus caballus przewalskii, which disappeared from the wild half a century ago (Der Sarkissian et al., 2015).

As a continuation of these successes, we can cite examples of the comparison of genomic diversity of inbred with outbred populations of the bull Bos taurus, comparisons of F1 descendants (from crosses between them) and descendants of F1 backcrosses with parental populations, as well as similar comparisons for the bison (Bison bison). The results of these studies independently confirm the finding of a decrease in the inbreeding degree when inbred strains of animals are crossed with their outbred relatives (Cronin, Leesburg, 2016). Finally, through the deciphering of the genome in the Austrian Fleckvieh bull Bos (primigenius) taurus, geographic locations influencing sperm quality were identified, and interbreeding options were found that improve this quality (Ferencakovic et al., 2017).

An increase in mortality from infections, as, for example, at the beginning of the reintroduction of Przewalski’s horses, is a much less studied parameter of the negative anthropogenic impact on animal populations (Robert et al., 2005).

Besides, during the creation of a reserve population of the Siberian grouse Falcipennis falcipennis, which had been on the verge of extinction in natural habitats, the intestinal microbiota of these birds changed, acting as a stressor of the immune system (Konyaev et al., 2013). An analysis of phylogenetic inertia of the infection–host network revealed an increase in the number of common infections of humans and domestic animals with the growing number of new tamed animals; this increase may be an epidemiological bridge connecting the anthropogenic environment with wildlife (Morand et al., 2014).

Finally, a possible counterargument to the above notion of a decrease in the reproductive potential of animals under the influence of humans is the domestic pig, which surpasses the wild boar in sperm quality (Almeida et al., 2006). The reason is selection for fertility for the sake of meat. Another counterargument is an increased proportion of females among domestic chickens in comparison with wild chickens as a consequence of selection for egg production (Zhang et al., 2020).

All of the above means that the decrease in reproductive potential during the domestication of new economically valuable species of animals (for example, the Asiatic wild ass Equus hemionus hemionus (Soilemetzidou et al., 2020)) can be compensated either by artificial selection for fertility in addition to the main desired trait or through interbreed crosses. When natural habitats of wild animals are included into economic land rotation by humans, an inbreeding-related diminution of their reproductive potential takes place (up to extinction), which can be compensated by subspecies crossings of these animals and by methods of assisted reproductive technology.

## Conclusion

We examined 68 human genes (see Table 1 and Supplemental Material) and 3080 DEGs of domestic animals versus their wild congeners. We found that the anthropogenic impact during the domestication of animals usually changes the expression of their genes in the same direction as seen in the expression alterations of orthologous human genes that worsen reproductive potential. By contrast, the natural habitat of wild animals maintains the intraspecific variation of expression of their genes in a way that equally corresponds to decreases and increases of reproductive potential in people, according to the expression alterations of the orthologous human genes.

## Conflict of interest

The authors declare no conflict of interest.
